# Reflecting on what? The difficulty of noticing formative experiences in the moment

**DOI:** 10.1007/s40037-018-0486-x

**Published:** 2018-11-12

**Authors:** Cheryl L. Holmes, Maria M. Hubinette, Malcolm Maclure, Harry Miller, Daniel Ting, Greg Costello, Melanie Reed, Glenn Regehr

**Affiliations:** Faculty of Medicine, University of British Columbia, Vancouver, B.C. Canada

**Keywords:** Health professional education, Reflection, Professional identity formation

## Abstract

**Introduction:**

In the spirit of enacting an educational model of guided, collective reflection to support positive professional identity construction in healthcare learners, we implemented a reflection-based course for medical students transitioning to clerkship with three goals: to sensitize learners to the hidden curriculum; to provide a safe and confidential forum to discuss their experiences; and to co-construct strategies to deal with the pressures in the clinical environment

**Methods:**

We used a design-based research protocol. Twelve students participated in ten sessions starting during their transition to clerkship. Faculty debriefed after each session, adjusting the format of the subsequent sessions. Data included student logs, transcripts of the course sessions, faculty debriefings, and the course evaluation. Data were analyzed via an iterative process of independent coding and discussion.

**Results:**

The main adjustments to the course were to eliminate didactic content in favour of using prompts prior to course sessions and de-emphasizing written reflection. Participants felt the course achieved its three goals and students reported enhanced resiliency during transition to clerkship, although, despite prompting, students offered no examples of their joining in with the negative behaviours around them.

**Conclusions:**

The course was successful in its key objectives. However, a key aspect of reflection, students noticing their own behaviour in the moment as something that needs to be reflected on, was challenging. Future research exploring the value of reflection as an intervention to redress the unwanted aspects of the hidden curriculum might focus on efforts to move the students to explicitly explore the enculturation process in themselves.

## What this paper adds

While the process of reflection has received increasing attention in medical education, the content of students’ reflections has been less fully explored. In addition to describing our own course to support the process of collective and guided reflection, we identified that the reflections our students brought to the group discussions were dominated by their observations of others’ behaviours rather than their own experiences of struggling with the pressure to conform. Thus, this paper, raises questions about how this selective identification of moments on which to reflect might undermine the value of guided, collective reflection for recognizing and resisting one’s own enculturation.

## Introduction

The negative influence of the hidden curriculum on the professionalization of medical students is well documented [1]. There are numerous reports suggesting that the ubiquitous experiences of mistreatment and negative role modelling [2] can undermine humanistic tendencies [3, 4] and lead to the adoption of undesirable behaviours and attitudes [5] that perpetuate a culture of unprofessionalism in medicine. Recognizing the limited success in trying to ‘fix’ it by changing the culture of medicine [6] or rooting out poor role models [7], there is an increasing focus on developing strategies to help students become intelligent consumers of the hidden curriculum [8]. At the core of this effort is the assumption that through deliberate reflection, medical students can become aware of the forces conspiring to influence them and can make intentional choices about their own development as healthcare professionals [8, 9]. However, authors have raised concerns about the use of unchecked reflection [10], which can often lead to the justification of suboptimal behaviours [11], and a subsequent shifting of attitudes to be in line with those suboptimal behaviours [12]. Thus, there is also an increasing recognition of the importance of guided self-reflection [13] to support learners in developing a positive professional stance. Moreover, as Holmes and colleagues have pointed out [8], for effective guided reflection to occur, students must notice the critical moments of pressure to conform in their daily experiences and be willing to bring these moments forward for collective discussion in a subsequent, safe and supported environment. But whether, and how, students might do this remains largely unexplored.

In an effort to both enact and further explore these ideas, from 2015 to 2017 we piloted GRAPHiC (Guided Reflection and Professionalization/Hidden Curriculum) based on the four-step curriculum proposed by Holmes and colleagues [8]: 1. *Priming—*educating students about the pressures of the hidden curriculum and the human propensity to conform or comply; 2. *Noticing—*encouraging students to be aware of their motivations and actions in situations where they experience pressures to conform to concerning practices; 3. *Processing—*guiding students to analyze their experiences through collaborative exercises designed to support reflection; and finally 4. *Choosing—*supporting students in selecting behaviours that validate and reinforce their aspirations to develop their best professional identity. The purpose of the current study was to evaluate the effectiveness of this course and to learn about the theory of reflection that served as the foundational premise of the course.

## Methods

### Research design

This was a design-based research study [14], aimed at developing, testing and refining both theory and practice, in continuous cycles of design, evaluation and redesign. Consistent with the tenets of design-based research, we based the development of our educational intervention on the four-step model of Holmes and colleagues which was evolved through an elaborate theoretical analysis of the literature [8]. We conducted our study of the intervention in an authentic context, we used multiple data sources and involved a variety of stakeholders in the design and evaluation of the course, and we iteratively altered the course based on ongoing evolving considerations of our theory-based assumptions in practice [14]. In this project, our intention was to test and refine both the GRAPHiC course *in situ* and theories regarding the place of reflection in medical student professionalization.

### Participants

Following institutional research ethics board approval, second-year medical students at one distributed site of our medical program were recruited by email and a subsequent information session. The first 12 students (from a potential of 26 students) who volunteered were enrolled in the course. All consented to the study aspect of the pilot in person during the first session. Course facilitators included three faculty from the undergraduate medical program, one fourth-year medical student and one first-year resident.

### Curricular intervention

The GRAPHiC course started during orientation week to clinical clerkship in June 2015 and spanned over Year 3 of clerkship with a follow-up session in Year 4 (2017). Ten 2‑hour facilitated small group (GRAPHiC) sessions (nine in Year 3 and one in Year 4) were conducted at intervals of 4 to 6 weeks, including an orientation (G1) and evaluation session (G6). The three objectives of the course were: 1) To ‘un-hide’ the hidden curriculum in clerkship; 2) To provide a safe and confidential forum for students to discuss their enculturation experiences with peers and guides; and 3) To collectively co-create strategies that students could utilize when they noticed these moments of pressure to conform in the future. Based on the theoretically derived model of Holmes et al. [8], it was hoped that these objectives would support the overall goal of helping students resist the moments of enculturation that perpetuate the hidden curriculum and thereby support their development of a positive professional identity.

The initial group meeting included a 1-hour didactic session to introduce the concept of the hidden curriculum as well as the constructs of compliance and conformity [15], followed by discussion with examples from the personal realm and clinical workplace in order to sensitize students to their own experiences of acceding to peer pressure. The facilitators were instructed to not be prescriptive regarding what constitutes professional (or unprofessional) behaviour, and to avoid using the course sessions as ‘whistle-blowing’ forums. Based in early feedback from students, subsequent sessions eliminated the planned didactic content to focus on open-ended discussion, inserting the learning objectives from the previously planned didactic content throughout the discussions instead. Each session integrated the four steps: *priming* (by relating stories from students and facilitators), *noticing* (by encouraging students through email prompts prior to the sessions), *processing* these stories and descriptions through small group discussions and *choosing* or co-constructing strategies together imagining what should and could have been done. After each session of the course, the facilitators debriefed, reflecting on the goals and approaches to support better reflection, and redesigned future sessions of the course.

Between curricular sessions, the students were asked to take field notes during their daily activities. Through email reminders and verbal prompts, students were coached to specifically note and journal their own process of enculturation. *In particular, they were asked to notice moments when they were experiencing dissonance; pressures to comply or conform to behaviours that moved them away from their best professional identity.* A secure online platform (Blackboard Connect^TM^) was provided for students to upload their journal entries to an e‑Portfolio where one facilitator had access to each student’s entries and posted replies and queries to the student.

After five sessions (G1–G5), a course evaluation (G6) was conducted by a faculty member not involved in the study in order to engage in additional midcourse corrections as needed. Three sessions (G7–G9) took place in Year 3 after the course evaluation, and a final session (G10) was conducted in the students’ final year of clerkship to discuss the impact of the course on their behaviours in Year 4.

### Data collection

Both the course sessions and facilitators’ reflections immediately after each session were audio-recorded and transcribed. Students’ stories from their personal e‑Portfolio journals were entered (with student consent) into the dataset for separate analysis by one investigator (to maintain student confidentiality). Data for the course evaluation after session 6 were obtained by mixed methods: facilitator and student surveys (quantitative and qualitative), as well as a student focus group (SFG) and a facilitator focus group (FFG).

## Data analysis

The qualitative datasets were entered into NVivo^TM^ Version 11 (Doncaster, Australia). Data analysis involved both deductive and inductive interpretation of the data. Deductively, we started with preliminary codes based on our theoretical framework for reflection (priming, noticing, processing and choosing) [8]. Three members of the research team coded the transcribed data after each session, evolving our preliminary codes as appropriate. Additionally, coders inductively developed and tested new codes as new ideas that informed our theoretical understanding of the phenomenon of guided reflection emerged. Preliminary findings were discussed with course facilitators and co-investigators. Discrepancies in interpretation were resolved by discussion and consensus building. The inductively developed themes were reconciled with previous deductively generated themes as the evolving thematic framework was further tested and explored. We presented our preliminary findings to the students periodically to member-check our interpretations of the data. Transcripts were re-read by one investigator to search for exceptions to our interpretations.

## Results

Below we report on both the effectiveness of the course itself and what we learned about the processes of reflection.

### GRAPHiC course evaluation data

All 12 students reported being ‘satisfied’ or ‘very satisfied’ with almost all aspects of the course including: the relevance of the course to their Year 3 experience; the facilitators’ understanding of what to expect of Year 3 students entering the GRAPHiC course; and the facilitators’ responsiveness to their questions. The course sessions were well attended and well received by students. They described looking forward to the group sessions and sharing their experiences with the other students and with the facilitators. Moreover, there was evidence that the three primary objectives of the GRAPHiC course were achieved.

*Un-hiding the hidden curriculum.* As hoped, students reported an openness to exploring the unintentional forces that were shaping their attitudes and behaviours as physicians. Moreover, they seemed remarkably amenable to the idea that they, as individuals, might be vulnerable to these pressures. As one student noted in the second session:

… *it’s funny you’re right, it’s almost like they’re this club and I’m showing that I’m not a part of it by wearing my mask or something. I don’t know, it’s kind of silly, but—yeah*. (G2)

*Creating a safe environment.* There were consistent reports that the course provided a safe and confidential forum for students to discuss their experiences with their colleagues and the facilitators, with students in the course evaluation focus groups offering statements such as:

*It felt like a safe place to discuss and reflect on our experiences as a third year. The best part was realizing that everyone else is having similar experiences.* (SFG)

That said, we will note below some data that might raise questions about the extent to which this was, in fact, true in critical ways.

*Co-constructing understanding and strategies.* Students found the group setting helpful to collectively co-create strategies based on sharing their experiences. Student comments in this regard centred around the effectiveness of the group process:

*I find it’s more meaningful to me to reflect and have input from other people, rather than reflecting and then not really getting any feedback or other input on the situation and how it was. … And so it was nice to hear what other people had to say*. (SFG)

Further, in their final year of clerkship, the students reported that the course impacted their processes of reflection. One student stated:

*This was, like, the only place in third and fourth year that was dedicated to … talking about sort of what you’re learning and what you’re noticing. And how those things are impacting you. I think that was really important. Yeah. Without it, I think it would be different*. (G10)

*Resilience. *In addition to these specific goals, students reported an unanticipated benefit; a great sense of relief that others were having the same challenging experiences and this helped support their own personal resilience. As one student described:

*I think I was really excited for third year, and then I was kind of disappointed at some of the things I saw, so it was a good group and avenue to have—just to kind of chat that out. And also, I think the biggest thing that I took away from this is that everyone’s kind of going through the same thing. And I found that really comforting*. (SFG)

### Lessons learned about the processes of reflection

The formal evaluation results outlined above suggest that the program was highly valued by students and facilitators alike. However, analysis of the course sessions, e‑Portfolio entries, and co-facilitator debriefings produced several insights that might raise some questions regarding the success of the program as envisioned from a theoretical perspective.

The first area of concern related to the use of journaling as a tool for students to document their experiences in the moment for later reflection. Our goal with the e‑Portfolio was to provide a mechanism by which students could document experiences in the moment for later reflection. Facilitators noted the absence of documentation in the e‑Portfolio and tried to encourage more use of the tool through verbal prompts and email reminders throughout the year. Despite these efforts, journal entries uploaded to the website were infrequent in early clerkship and then tapered off to almost none by mid-clerkship. In discussion, students acknowledged their unwillingness to use the e‑Portfolio offering a variety of explanations related to competing demands:

*I’m having a hard time just, like, eating and showering and stuff right now … and oh, my gosh, like, dictations, you name it. … But yeah, I hope I get a little more sorted in the next couple of weeks. Usually I’m pretty good at this type of stuff, but—yeah, it’s just the timing.* (G2)

as well as to difficulty with the writing process itself:

*Yeah, so I wrote two under the personal section. One of them I just started writing back in June and then I had gone away and then it was kind of, like, I needed to do some research for it. And on the second one I wrote right away in my first week. But I guess whenever I’ve done reflections in the past they’ve always been very long, and I felt that this one was really short. So, I thought I would come back to it, and then I just never did.* (G2)

Consistent with the faculty observations, the one area of clear dissatisfaction among the students was the e‑Portfolio; 7 of the 12 students (59%) were neutral or disagreed that this online aspect of the course was relevant and useful in supporting their learning.

As a second, related, domain of concern, facilitators noted that the stories students chose to share and discuss all tended to have an external focus. Students readily described egregious behaviours in other healthcare professionals, such as denigrating other colleagues, speaking disrespectfully when referring to patients, and making jokes with racist and sexist overtones. However, to the extent that they described their own role in these situations, the descriptions focused only on their ‘passive’ complicity. As exemplified by one participant’s journal entry, *‘I have to admit that although I was not actively participating that I didn’t actively object either’.* (journal entry). For the duration of this course (the entire clerkship year), across ten course discussions, and in all the journal entries, there were no examples of students initiating or even ‘joining in’ with behaviours that they were identifying in others. Concerned that students would not be able to notice themselves adopting negative attitudes and behaviours of those around them, facilitators specifically prompted and questioned students during sessions and by email, but no students reported actively participating in any problematic behaviours. When pressed, students acknowledged this gap. One student attributed this to the subtlety of the moment:

*In the moment, it’s very difficult to notice when I am conforming because for me this often happens subconsciously, and it is not until afterwards that I am able to notice these changes in my behaviours or thoughts.* (journal entry)

Another suggested that it was more about the difficulty of documenting in the moment:

*For me that I find that I—like, sometimes I would think—like, in the back of my mind to notice these things and often I do. But by the time there is time to write it down, the momentum’s lost. I’m, like, oh, well, I don’t feel like—like, I’ve already conformed or I’m already—like, that momentum is lost when I get to writing it down. I end up not writing it down.* (G2)

Despite this acknowledgement, specific examples of the students instigating or simply joining in were not raised during these or subsequent discussions, which raises important questions regarding the theoretical premise of the course and possible limitations on its success.

## Discussion

Based on the formal evaluation process, the GRAPHiC course could be deemed a success. Students described the course sessions as a safe environment to raise and discuss troubling experiences in clerkship and indicated that the discussions were helpful both for reinforcing their values and for developing strategies to safely preserve those values in the clinical context. Encouraged by this success, we have now incorporated the elements of the course in an expanded program across the four sites of our distributed program. Groups of eight students are followed longitudinally by a coach over their 4 years of medical school, with the purpose providing a safe, supported space to intentionally develop their professional identity. Building on our findings from this pilot, the small group sessions have integrated didactic content into active discussion within each session and the program emphasizes guided group discussions based on clinical experiences over written self-reflections.

That said, a core theoretical premise underpinning the course was that, for effective guided reflection to occur, one must notice the critical moments of pressure to conform and bring them forward for discussion. Consistent with the findings of others [4, 16], the students in our pilot study certainly noticed the unprofessional behaviours of others and were able to recount and explore them afterwards. Further, while many of the examples students raised were accompanied with a clear rejection of the behaviour, some of their recounted experiences suggested that they were sensitive to the implicit social pressure to join in with these behaviours, and we were able to leverage these experiences into meaningful conversations and reflections with the students. Interestingly, however, despite our best efforts, we were unable to entice students to offer examples of their own active participation, however minor, in these activities.

There are several potential explanations for this finding. First is the possibility that we were studying students too early in their developmental trajectory and the maladaptive behaviours we were trying to curtail occur later in the enculturation process than we had originally predicted. Interestingly, on re-examining the literature with this question in mind, we found no studies that offered insight into this trajectory. With few exceptions [5], the studies we did identify tended to describe student reports that focus on their senior colleagues (or other professions). So, it remains an open question as to when (and how) these behaviours start to manifest as observable acts of commission.

A second obvious possibility is that, despite their assertions that they felt safe to discuss sensitive issues, students may have noticed themselves participating in unprofessional behaviour but were unwilling to bring them forward to the group. This raises questions about whether it would ever be possible to create a sufficiently safe collective reflection environment within the confines of formal curricular structures to enable such ‘confessions’ [17]. If not, this raises doubts about the place of formal exercises designed to support reflection (even more so exercises that require formal written documentation such as portfolios) in supporting students’ efforts to come to terms with the conditions of social pressure to which they discover themselves to be particularly prone. Of course, this might have been a unique shortcoming of our own efforts to create sufficient safety. If so, however, the shortcoming would not have been obvious to us based on the students’ descriptions during the program evaluation process, which suggests a possible lacuna when assessing the effectiveness of such efforts in similar programs.

A more concerning possibility is that these small lapses in professionalism were so subtle as to defy noticing, resulting in a progression toward unprofessional behaviour that is akin to ‘death by 1,000 cuts’. Even if the action is noticed in the moment, it may be that without some form of intentional ‘metacognitive tag’ to the event, students lose track of the experience. If so, the event and its associated emotions and cognitions might not be easy to recall later, and therefore these critical moments might very quickly become subliminal and unavailable for intentional reflection. Thus, perhaps it is the *less* egregious behaviours that are more ‘dangerous’ in terms of being formative and enculturating students because they are so hard to notice. Others have provided evidence that ‘habituation to dilemmas’ does occur and becomes internalized [19]. This may place important limitations on the value of self-regulation in resisting this process. It would suggest the need for a shift in culture toward a more ‘collective responsibility’ for professional behaviour [20] and enabling workplace conversations that support this process.

We have also been considering reasons why the e‑Portfolio was unsuccessful as a mechanism for documenting transient moments of enculturation. The students’ comments from the course evaluation offer some intriguing possibilities. First it may be that even ‘personal’ reflections, when written down, act as a form of legacy document, an artifact that can be accessed later, but not always suitable for exploring a potentially shame-inducing experience. Second, our students seemed to be articulating the limits of the written word in capturing the nebulous feelings they were experiencing. In part, this seemed to be because the ‘right’ word was not available and in part because the structure of the written word did not fit the ongoing processes of reflection, because it forced a linearity of structure on ideas that were more iterative and circular. Tannen has discussed oral versus written discourse, citing the interpersonal focus of orality, and the limitations of the written word stating that, ‘print is a great leveler; it reduces or inflates all utterances to lines of equivalent evaluative status on a page’ [18]. To the extent that these possibilities (extrapolated from our students’ expressions of reluctance) are in play, this has implications for the use of written reflections in e‑Portfolios in that, if we force students to engage in such activity we may be problematically distorting the processes of reflection itself both through the process of turning reflection into a performative act and through the linear structure of the written word.

While this is a small study in a single academic program, and the recruitment of study participants was voluntary and may have created a selection bias in our results that does not represent the larger medical class, our experience does highlight that a key aspect of the processes of reflection, noticing our own behaviour in the moment as something that needs to be reflected on, may be more challenging than current models of reflection assume. We suggest, therefore, that programs exploring the use of portfolios and formalized group reflection as a mechanism for enhancing positive professional identity formation might do well to attend to the question of what is being reflected on.
